# Research on a Burn Severity Detection Method Based on Hyperspectral Imaging

**DOI:** 10.3390/s25051330

**Published:** 2025-02-21

**Authors:** Sijia Wang, Minghui Gu, Mingle Zhang, Xin Tan

**Affiliations:** 1 Department of Burn and Plastic Surgery, Jilin Provincial People’s Hospital, Changchun 130021, China; wangsijia0726@163.com; 2 Changchun Institute of Optics, Fine Mechanics and Physics, Chinese Academy of Sciences, Changchun 130033, China; zhangmingle21@mails.ucas.ac.cn; 3 University of the Chinese Academy of Sciences, Beijing 100049, China

**Keywords:** burn detection, hyperspectral imaging, Mamba, bidirectional scanning, machine learning

## Abstract

The accurate detection of burn wounds is a key research direction in the field of burn medicine, as diagnostic results directly influence the risk of wound infection and the formation of hypertrophic scars. Currently, burn diagnosis is primarily dependent on the clinical judgment of physicians, but its accuracy is typically only between 65% and 70%. Therefore, a non-invasive, efficient method for burn severity assessment is urgently needed. Hyperspectral imaging (HSI), as a non-invasive and contactless spectral detection technique, has been shown to precisely monitor structural changes in burn-affected skin tissue and holds significant potential for burn depth diagnosis. However, research on the application of burn severity detection remains relatively limited, which restricts its widespread use in clinical settings. A burn severity detection classification network (MBNet) based on the Mamba model is proposed in this paper. Through a bidirectional scanning strategy, MBNet effectively captures the long-term dependencies of spectral features, accurately establishes the relationships between bands, and efficiently distinguishes subtle spectral differences under different burn conditions. MBNet provides a reliable and efficient method for clinical burn severity assessment. A comparison of MBNet with seven typical machine learning algorithms on a custom dataset demonstrates that MBNet significantly outperforms these methods in terms of accuracy.

## 1. Introduction

Burns are injuries to the skin and other tissues caused by factors such as high temperatures, radiation exposure, electrical current, friction, or chemical substances [1]. Approximately 11 million people worldwide suffer burns each year, with burn injuries ranking fourth among all injury types. The assessment of burn wound depth and the determination of healing potential are critical aspects of clinical treatment [2]. Currently, burn depth diagnosis mainly relies on the experience of clinicians. However, due to the loss of skin tissue functionality caused by burns, visual observation is limited, and even experienced physicians can achieve only 65–70% accuracy in clinical evaluations [3]. Although tissue biopsy is considered the “gold standard” for diagnosing burn depth, it has not been widely adopted in clinical practice due to its time-consuming nature, high cost, invasiveness, and limited sample size [4]. Therefore, the development of an efficient, precise, and non-invasive method for burn depth diagnosis is considered especially necessary.

Various non-invasive techniques have been used to assess burn severity, such as in vivo staining detection, thermal imaging, ultrasound imaging, magnetic resonance imaging (MRI), and laser Doppler technology [5]. In comparison, hyperspectral imaging technology, with its advantages of broad, rapid, and non-invasive data acquisition, has become an effective tool for evaluating the degree of skin burns. Hyperspectral images (HSIs) contain rich spectral information, and many typical feature learning methods, such as K-nearest neighbor (KNN) [6], Support Vector Machine (SVM) [7], Random Forest (RF) [8], Gradient Boosting Machine (GBM) [9], and Linear Discriminant Analysis (LDA) [10], have been widely applied to feature extraction and hyperspectral data analysis. However, HSIs often contain hundreds of spectral channels, and these high-dimensional features significantly increase computational complexity, with traditional methods prone to the “curse of dimensionality”, reducing the generalization ability of learning models. Although RF and GBM can handle high-dimensional data through feature selection, effectively selecting the most relevant features remains a challenge. Although traditional dimensionality reduction techniques aim to reduce data dimensions while retaining essential information, they may still result in the loss of certain spectral details. In recent years, deep learning-based hyperspectral data analysis methods have gained increasing attention [11]. Deep learning models, such as Convolutional Neural Networks (CNNs) [12] and Recurrent Neural Networks (RNNs) [13], can automatically extract spectral features from HSIs at multiple levels and scales, reducing the loss of detailed information. These methods not only significantly improve classification accuracy but also exhibit high stability and adaptability across different burn depths and skin types. However, these methods still struggle to effectively capture the long-range dependencies of spectral features, and their ability to analyze hyperspectral data in complex scenarios has not been fully utilized, leaving room for improvement in spectral classification accuracy.

The recently proposed Mamba model [14] is based on the state-space model (SSM) [15] and improves the structured SSM (S4) [16] by introducing a selection mechanism. The Mamba model is capable of effectively capturing long-range dependencies, has higher computational efficiency than the Transformer [17], and has shown great potential in natural language processing tasks [18,19]. In this paper, we propose a Mamba-based classification network (MBNet). The network adopts a dual-branch architecture that facilitates bidirectional band-level information extraction and overcomes the limitations of Mamba’s unidirectional modeling. With this design, MBNet can effectively establish relationships between bands and accurately distinguish subtle spectral differences across various burn conditions, providing a reliable method for the clinical assessment of burn severity.

## 2. Related Works

### 2.1. Application of HSI in Burn Assessment

HSI is an imaging technique that combines both imaging and spectral technologies. It typically covers a continuous portion of the spectrum, enabling the scanning of hundreds of spectral bands across ultraviolet, visible, infrared, and even mid-to-far infrared wavelengths [20]. This provides both two-dimensional spatial information and one-dimensional spectral information. With this technology, the reflectance of each pixel in an HSI can be measured. Compared to traditional RGB and grayscale images, HSI offers more spectral bands and higher spectral resolution, enabling the detection of object changes that cannot be captured by conventional imaging technologies, especially in identifying subtle spectral differences under different pathological conditions [21].

HSI is widely applied in various fields such as Earth observation, geology, vegetation science, and soil science [22,23]. In the medical field, Anselmo et al. [24] were the first to propose multispectral analysis, involving the reflection of red, green, and infrared light, to assess burn depth. The basic principle of this method lies in the light attenuation signals caused by blood absorption of different spectra, which can be used to differentiate the severity of burns. Eisenbeiss et al. [25] proposed an objective burn depth assessment method based on multispectral imaging, enabling non-specialist physicians to assess burn depth in the early stages. Through practical research, Mihaela et al. [26] demonstrated the value of HSI in characterizing burn wounds, highlighting that a lack of light absorption around 670 nm may be a key characteristic of burns, which could be explained by the absence of absorption components such as keratin. Wang et al. [27] integrated hyperspectral technology with machine learning, establishing a regression model based on burn data collected using fiber optic spectrometers, successfully achieving the quantitative detection of burn depth.

Deep learning, which is based on deep neural networks, is an end-to-end network model that has advantages in image processing. Compared to its applications in other fields, deep learning still has significant potential for development in medical HSI processing. Huang et al. [28] combined a CNN model with Gabor filters to enhance blood cell classification under small-sample conditions. Sommer et al. [29] utilized residual neural networks (ResNets) to classify kidney units. Wang et al. [4] proposed a burn detection method based on HSI and deep transfer learning, which overcame distribution discrepancies between different datasets by introducing deep transfer learning. Zunair et al. [30] employed adversarial training and transfer learning to automatically classify skin lesion images, thereby resolving the issue of class imbalance and achieving experimental performance comparable to that of expert dermatologists. Additionally, many studies have used CNNs for cancer cell classification [31,32].

In the field of medical imaging, the introduction of deep learning methods has gradually revealed their unique advantages. Many of the latest deep learning techniques have been borrowed from other fields and successfully applied in medical imaging, yielding excellent results [33]. The recently proposed Mamba model [14] is based on the SSM [15], offering strong remote modeling capabilities while maintaining linear computational complexity, although it cannot be directly applied to HSI due to spectral continuity. Li et al. [34] were the first to introduce an image-level HSI classification model based on Mamba, showcasing the tremendous potential of Mamba as the next-generation backbone for HSI models. Ma et al. [35] combined mobile-friendly convolution with Mamba to construct a lightweight backbone from the perspective of frequency decoupling, achieving impressive performance across multiple downstream tasks. This motivated us to apply the Mamba model to burn severity detection tasks, as it not only maintains linear computational complexity but also effectively models long-range dependencies, which is expected to enable rapid and accurate clinical assessment of burn severity.

### 2.2. Mamba Model

**Figure 1:**
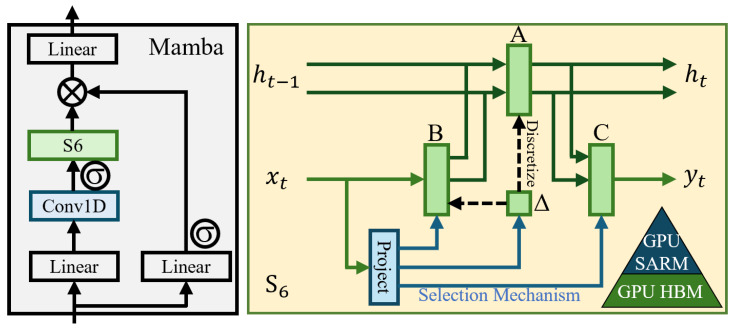
Structure of the Mamba block.

The traditional SSM [15] primarily relies on linear time-invariant simplifications, offering the advantage of linear time complexity. However, it struggles to effectively capture the contextual information in input sequences. To address this issue, Mamba [14] introduces a selective mechanism and proposes the selective SSM (S6) to enable dynamic interactions between sequential states. Unlike the traditional static parameterized SSM, in which matrix parameters are fixed, S6 dynamically generates its matrix parameters based on the input data. This allows the model to adaptively adjust based on the content of the input sequence, thereby better modeling the contextual relationships within the sequence and enabling selective attention to each sequence unit. This selective mechanism provides the Mamba model with additional flexibility when processing sequential data, allowing it to selectively retain or discard information based on the relevance of each input. Additionally, Mamba introduces a hardware-aware algorithm to improve computational efficiency and practical performance. The specific structure of the Mamba block is shown in Figure 1.

## 3. Proposed Method

### 3.1. Network Architecture

**Figure 2:**
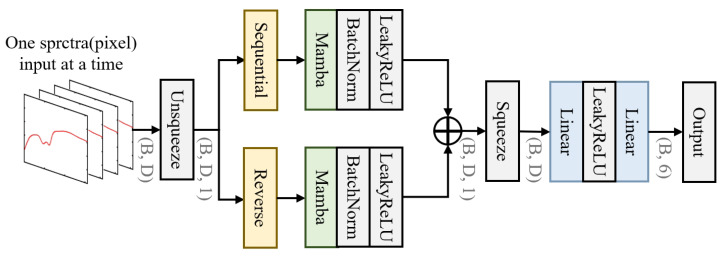
The dual-branch spectral feature extraction network.

To fully extract valuable information from the spectral data and establish an accurate, real-time burn assessment and classification method, we propose a novel dual-branch spectral feature extraction network. The specific architecture is shown in Figure 2. This network, referred to as MBNet, is composed of a bidirectional Mamba global spectral feature extraction module and a feature compression classification module, which are cascaded.

In hyperspectral classification tasks, the model needs to precisely distinguish subtle spectral feature differences between different pixels. Therefore, the Mamba module was introduced as a fundamental unit to model long-range dependencies. The Mamba module was designed to operate in specific directions during data processing and has demonstrated strong global context modeling capabilities in natural language processing tasks. However, when handling hyperspectral data, especially in complex spectral correlation scenarios, existing Mamba methods often struggle to efficiently extract and integrate spectral features. To overcome this limitation, a bidirectional Mamba global spectral feature extraction module was designed to comprehensively capture the spectral information in HSIs.

In the spectral feature extraction module, the Mamba module performs bidirectional modeling along the spectral feature dimension. By deeply exploring the dependencies between spectral features, Mamba is able to effectively update and enhance the representation of spectral features, thus improving the accuracy of spectral classification. Let $Y\in R^{N\times D}$ represent the set of spectral data to be tested, where *N* is the number of target pixels and *D* is the number of spectral bands. The target $Y_{\mathrm{in}}\in R^{B\times D}$ is input into the network in batches, where *B* denotes the batch size, i.e., the number of spectral curves fed into the network at each iteration. The input spectral data $Y_{\mathrm{in}}$ undergo a dimensional padding operation to meet the Mamba module’s scanning dimension requirements along the spectral sequence. The padded data are then fed into the Mamba module for processing, as shown by the following formulas:

(1) $$Y_{\mathrm{out}\text{-}\mathrm{seq}}=\mathrm{LeakyReLU}\left(\mathrm{BatchNorm}\left(\mathrm{Mamba}\left(Y_{\mathrm{in}\text{-}\mathrm{seq}}\right)\right)\right)$$

(2) $$Y_{\mathrm{out}\text{-}\mathrm{rev}}=\mathrm{LeakyReLU}\left(\mathrm{BatchNorm}\left(\mathrm{Mamba}\left(Y_{\mathrm{in}\text{-}\mathrm{rev}}\right)\right)\right)$$

(3) $$Y_{\mathrm{out}}=Y_{\mathrm{out}\text{-}\mathrm{seq}}+Y_{\mathrm{out}\text{-}\mathrm{rev}}$$

The input feature $Y_{\mathrm{in}}$ is expanded along the spectral dimension in both the sequential and reverse directions, thereby overcoming the limitation of unidirectional modeling in the Mamba module. The BatchNorm layer normalizes the network parameters and intermediate outputs (using the batch mean and standard deviation), aiming to mitigate common issues such as exploding or vanishing gradients during training, thus accelerating the convergence process of the network. To avoid the “dead neuron” problem that may be caused by the ReLU activation function, the LeakyReLU activation function is chosen, ensuring that gradients can be effectively propagated and further improving network training performance. Through bidirectional sequence scanning, the Mamba module fully captures spectral contextual information. At the end of the module, the sequential and reverse sequence features are fused, enhancing the representational power of the features and further improving the classification accuracy of the model.

After feature extraction, the output feature $Y_{\mathrm{out}}$ needs to undergo further feature compression to meet the classification requirements. The specific process is as follows:

(4) $$\mathrm{Output}=\mathrm{Linear}\left(\mathrm{LeakyReLU}\left(\mathrm{Linear}\left(Y_{\mathrm{out}}\right)\right)\right)$$

In this process, the first linear layer compresses the spectral feature dimension to 100, while the second linear layer further compresses it to the number of categories (6 in this paper). To optimize the training process, cross-entropy loss is used as the loss function, and the network is trained using the backpropagation algorithm, ultimately achieving high classification accuracy.

### 3.2. Parameter Settings

During the training process, the Adam optimizer was employed with an initial learning rate of 1 × $10^{-3}$, and an exponential decay strategy was applied to adjust the learning rate, with a decay step of 10 and a decay rate of 0.9. The batch size was set to 2048, and the training consisted of 1000 epochs. All experimental code was written in Python 3.10, using PyTorch 2.1.1 as the deep learning framework. During training, gradient computations were performed on an NVIDIA GeForce RTX 4090 (24 GB) GPU.

## 4. Discussion

### 4.1. Dataset

**Figure 3:**
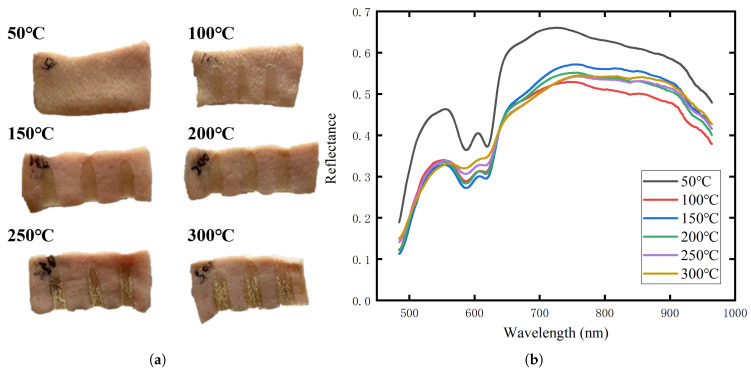
Experimental dataset: (**a**) Burn wounds on pig skin sample; (**b**) Spectral reflectance curves of various burn wounds.

**Figure 4:**
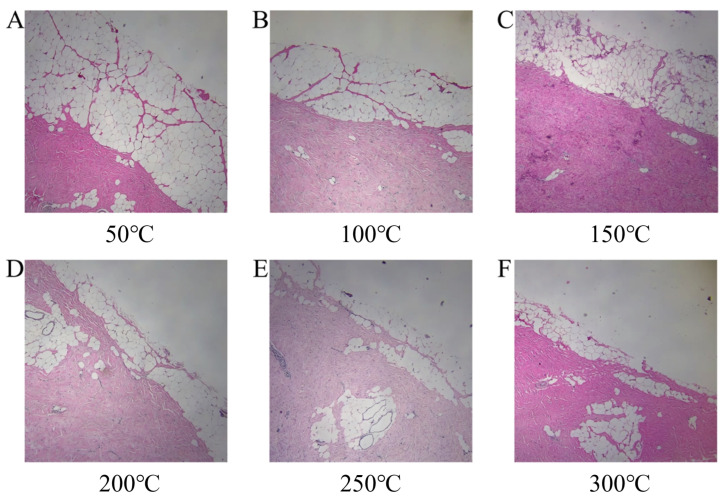
The pathological H&E staining results of the tissue sample at different temperatures are shown in sub-figures (**A**–**F**).

The skin of pigs is similar to human skin in terms of tissue structure and thermophysiological responses, making it an ideal model. In this study, fresh pig skin was used to simulate human burn tissue. The pig skin samples were obtained from a male Bama mini-pig approximately six months old and weighing around 18 kg. Prior to the experiment, the pig skin samples were heated in a water bath at temperatures ranging from 30 to 40 °C to simulate the normal skin temperature. The subcutaneous fat was then removed, and each sample was divided into six pieces. Each piece of skin was subjected to burning with an electric soldering iron, applying the same force at burn temperatures of 50 °C, 100 °C, 150 °C, 200 °C, 250 °C, and 300 °C for a duration of 6 s. Figure 3a shows the pig skin samples after burning under different temperature conditions. Following the burns, the samples and a standard reflectance plate were placed at the same height, and uniform illumination was provided using a halogen lamp light source. Data were collected using a scanning visible-near infrared imaging spectrometer with a wavelength range of 400–1000 nm and 260 bands. The collected data were used as the dataset for this study. The spectral reflectance curves at different burn temperatures are shown in Figure 3b, where distinct spectral interference can be observed, although noticeable spectral differences are still evident near 600 nm and in the near-infrared range. According to [26], the lack of light absorption in the range of 600–700 nm is one of the characteristics of burns, while the spectral features in the near-infrared range are related to the sum-frequency and second-harmonic absorption of some organic compounds [4], indicating the need for further processing with feature extraction algorithms. After data collection, the samples were fixed in a formalin solution and sent to the pathology department for burn depth measurements by professionals. Figure 4 shows the H&E-stained pathological sections of the tissue samples. As the burn temperature increased, the burn depth progressively deepened, further validating the effectiveness of the dataset.

### 4.2. Methods for Comparison

In this section, we perform comparative experiments with other common methods. The methods used for comparison are as follows:

1. KNN [6]: An instance-based learning algorithm that, given a test sample, finds the K-nearest neighbors in the training set and predicts the class or value of the test sample based on the classes of its neighbors.
2. SVM [7]: A supervised learning algorithm widely used for classification tasks, which aims to find a hyperplane that separates samples of different classes while maximizing the distance between the hyperplane and the nearest samples.
3. RF [8]: An ensemble learning algorithm that constructs multiple decision trees using the Bagging (Bootstrap Aggregating) method and generates the final prediction through voting or averaging.
4. GBM [9]: A gradient boosting algorithm that sequentially trains multiple weak learners (usually decision trees), with each new model correcting the errors of the previous one, thereby improving overall predictive performance.
5. LDA [10]: A supervised learning method that performs classification by finding the feature combinations that most effectively differentiate between different classes.
6. MLP [36]: A feedforward neural network composed of multiple layers, typically including an input layer, several hidden layers, and an output layer. Each neuron is fully connected to those in the previous layer, making it suitable for solving nonlinear problems.
7. 1D CNN [37]: A deep learning method that extracts local features from one-dimensional data through convolution operations, thereby building high-level representations of the data.
8. Transformer [17]: A deep learning architecture that uses the self-attention mechanism to process sequential data in parallel, enabling efficient capture of long-range dependencies and relationships.

Through a series of comparative experiments, we evaluated the performance of the different machine learning algorithms in the burn severity prediction task. The experimental dataset included spectral data collected at various temperatures (50 °C, 100 °C, 150 °C, 200 °C, 250 °C, and 300 °C), which captured the surface reflectance spectral characteristics at different burn severities. By using the spectral signals as input features, key information related to the burn temperature was effectively extracted, enabling accurate prediction of burn severity.

### 4.3. Quantitative Performance Metrics

To ensure the stability and reliability of the experimental results, the training process of all models followed the standard 10-fold cross-validation method. Specifically, the dataset was randomly divided into 10 subsets, with 9 subsets used for training the model and the remaining subset used for testing. This process was repeated 10 times, each time selecting a different subset as the test set. The final evaluation result was the average of the 10 experiments, reducing errors caused by random factors. In terms of performance evaluation metrics, three common metrics were used: Overall Accuracy (OA), Average Accuracy (AA), and Kappa.

OA is one of the most commonly used classification performance metrics. It represents the proportion of correctly classified samples among all samples. This metric evaluates the overall performance of the model by calculating its classification accuracy across the entire dataset. The formula for calculating OA is

(5) $$\mathrm{OA}=\frac{\sum_{i=1}^{N}{\mathrm{TP}}_{i}}{\sum_{i=1}^{N}{\mathrm{Total}}_{i}}$$

where ${\mathrm{TP}}_{i}$ is the number of correctly classified samples for class i, ${\mathrm{Total}}_{i}$ is the total number of samples in class i, and *N* is the total number of classes. Overall Accuracy reflects the average performance of the model across all classes, but it may be influenced by the dominant class in the presence of class imbalance, leading to an overestimation of the model’s performance.

AA evaluates the model’s performance by calculating the accuracy for each class and then averaging the accuracies across all classes. It is more effective than OA in situations with class imbalance, as it avoids the influence of the dominant class. The accuracy for each class is the proportion of correctly classified samples within that class relative to the total number of samples in that class. The formula for calculating AA is

(6) $$\mathrm{AA}=\frac{1}{N}\sum_{i=1}^{N}\frac{{\mathrm{TP}}_{i}}{{\mathrm{Total}}_{i}}$$

Kappa measures the consistency of the model’s classification results while accounting for the influence of random chance. Unlike OA, the Kappa coefficient not only considers the model’s correct classifications but also takes into account the likelihood of correct classifications occurring by chance. The formula for calculating Kappa is as follows:

(7) $$\mathrm{Kappa}=\frac{P_{o}-P_{e}}{1-P_{e}}$$

where $P_{o}$ is the overall classification accuracy of the model and $P_{e}$ is the expected agreement, computed as the sum of the product of actual and predicted values divided by the square of the total number of samples.

### 4.4. Comparison of Results

Table 1 presents the quantitative results of the various comparison methods. Overall, the proposed MBNet model achieved an average accuracy of 93% across the six burn-degree prediction tasks, significantly outperforming other conventional models. LDA is suitable for datasets with fewer categories or lower dimensionality and, therefore, performed relatively poorly in high-dimensional, multi-class burn-degree prediction tasks, yielding lower OA and AA values. KNN, RF, and GBM exhibited similar performance, with both classification and average accuracies around 83%. While RF and GBM excelled at handling non-linear relationships, KNN tended to suffer from the “curse of dimensionality” in high-dimensional data, resulting in inferior performance. Among traditional machine learning methods, SVM performed the best, benefiting from its ability to map data to high-dimensional spaces using kernel functions, thereby effectively handling non-linear data and identifying accurate decision boundaries. Due to the strong feature-learning capability of deep learning, several deep learning methods performed noticeably better than traditional machine learning approaches. The Transformer model, however, exhibited the weakest performance among deep learning models, likely because the spectral data were relatively simple, leading to model overfitting. Although Transformer models typically excel at handling complex patterns, they struggle to leverage their advantages when the data features are relatively simple. Compared to Transformer and MLP, 1D CNN excelled at extracting local features from spectral sequences, capturing local variations in spectral data more effectively, and yielding near-optimal results. MBNet improved upon this by performing bidirectional modeling along the spectral feature dimension, which better captured global dependencies between spectral features. By introducing bidirectional information flow, the method enhanced the learning of the global structure of spectral features, thereby achieving the best performance. The Kappa coefficient of the MBNet model was 0.9170, demonstrating high reliability in classification accuracy and consistency. In comparison, the Kappa coefficients of the other models were generally lower, further confirming that MBNet not only excels in classification accuracy but also provides greater stability and consistency in burn-degree prediction tasks.

**Figure 5:**
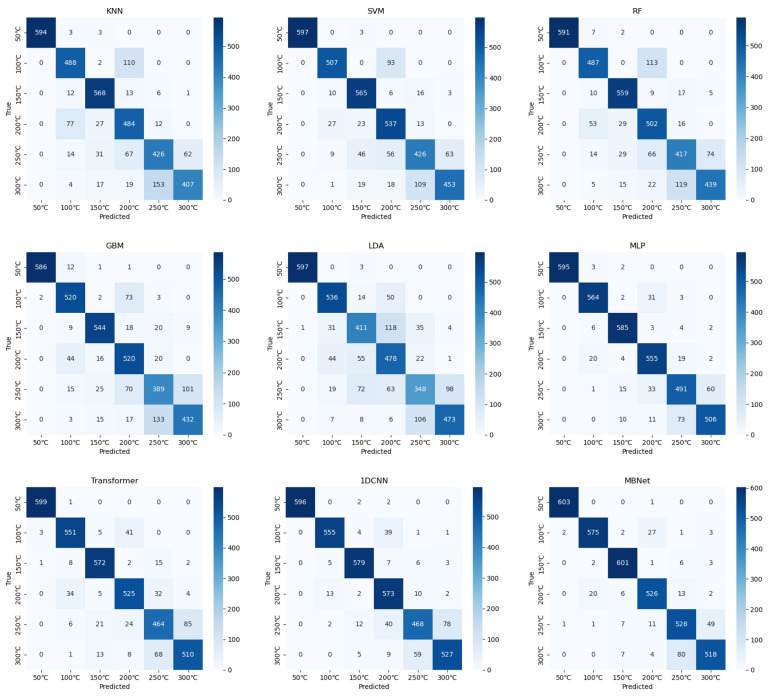
Confusion matrices of different methods.

Figure 5 presents the confusion matrices of various models, illustrating the performance differences among the models and further highlighting the advantages of MBNet in terms of accuracy, robustness, and handling complex categories. Compared to models such as Transformer, MLP, and 1D CNN, MBNet excelled at reducing misclassifications. In the case of burns at 50 °C, MBNet successfully classified 99.5% of the samples correctly. In high-temperature burn predictions at 250 °C and 300 °C, the misclassification rate of MBNet was notably lower than that of the other models, demonstrating its strong generalization ability. For categories with similar feature distributions, such as those at 150 °C and 200 °C, MBNet effectively reduced misclassifications, showing strong discriminative power and maintaining stability and balance across diverse scenarios. This is crucial for classification stability and accuracy in practical applications. MBNet captured subtle pattern differences in the data more effectively, showcasing its powerful feature learning capability. This excellent performance not only reflects MBNet’s advantage in feature learning but also highlights its ability to better extract meaningful features from the training data, reducing the risk of overfitting and misclassification while improving the model’s prediction accuracy and stability.

In conclusion, MBNet holds significant application value in burn severity detection, with advantages including higher classification accuracy, robustness to complex overlapping categories, and a remarkable ability to reduce misclassifications in temperature categories. Specifically, MBNet demonstrates significant performance improvements in accurately distinguishing between different burn temperature categories and enhancing diagnostic precision. The classification results show good balance across all temperature categories, enabling the model to effectively identify different stages of burns in real-world scenarios, preventing diagnostic delays or incorrect treatments due to temperature classification errors and thereby enhancing the reliability and accuracy of burn diagnosis.

### 4.5. Ablation Experiments

The proposed MBNet comprehensively captures the spectral information in HSIs through a bidirectional Mamba global spectral feature extraction module and compresses the features to meet classification requirements. To verify the effectiveness of the Mamba module, we conducted experiments to evaluate the impact of a single sequential branch, a reverse branch, and the removal of the Mamba module on the network’s performance. The results are shown in Table 2. The results show that when the Mamba module was completely removed, the network approximated an MLP architecture and exhibited performance similar to that of the MLP in the comparison methods. When only the unidirectional Mamba module was retained, the network’s overall performance slightly decreased but still outperformed the other comparison methods. However, when both bidirectional branches were retained, the limitations of unidirectional Mamba modeling were overcome, and the network achieved optimal overall performance. This result demonstrates the effectiveness of the bidirectional Mamba global spectral feature extraction module in burn classification tasks and highlights that the comprehensive extraction of spectral information is key to achieving highly reliable burn recognition.

### 4.6. Processing Time

The processing times of the different methods in the experiment are shown in Table 3. All methods were implemented in PyCharm (2022), and the experiments were conducted on a computer equipped with an Intel Core i5-13600KF processor, 32 GB of memory, and an NVIDIA GeForce RTX 4090 GPU. The gradient training of 1D CNN, Transformer, and MBNet was accelerated using the GPU, while the remaining methods were executed on the CPU. Due to the smaller number of parameters, the training times of several traditional methods were significantly shorter than those of deep learning methods. Among these, the instance-based learning method KNN only required storing the dataset during the training phase, resulting in almost zero training time, but it incurred a substantial computational cost during the prediction phase. The Transformer model, which exhibited quadratic computational complexity, experienced a notable increase in computation time. In contrast, the proposed MBNet demonstrated a linear complexity advantage in data processing and showed significant superiority in model prediction time, thereby enhancing the feasibility of this method in practical applications.

## 5. Conclusions

The accurate assessment of burn wound depth is of significant importance for optimizing treatment plans and improving clinical outcomes. Traditional clinical evaluation methods are often limited by their subjective nature and poor reproducibility. Hyperspectral imaging, as a simple, non-invasive, and efficient spectral detection tool, offers a viable solution to address this issue. However, in complex real-world application scenarios, the analytical capabilities of hyperspectral data have not been fully utilized, and the classification accuracy of existing models could be improved. This paper proposes a dual-branch feature extraction classification network named MBNet, which integrates the sequence modeling capabilities of the Mamba architecture to construct a parallel feature learning framework. This framework enhances the correlation analysis between spectral bands on a global scale, enabling the precise differentiation of subtle spectral variations across various burn temperatures. Nevertheless, the algorithm has yet to fully incorporate spatial information when analyzing hyperspectral data, which may limit its effectiveness in complex scenarios. Future research will explore the incorporation of spatial information into the algorithm to further enhance its accuracy and adaptability. This research not only contributes to advancing burn diagnosis technology but also provides new insights and methodologies for the early diagnosis of other dermatological conditions.

## Figures and Tables

**Table 1:** Comparison of quantitative results among different methods.

CLASS	KNN	SVM	RF	GBM	LDA	MLP	1D CNN	Transformer	MBNet
50 °C	100.00	100.00	100.00	99.66	99.83	99.83	100.00	99.34	99.50
100 °C	81.61	91.52	84.55	86.24	84.14	93.87	96.52	91.68	96.15
150 °C	87.65	86.13	88.17	90.22	73.00	90.05	95.86	92.86	96.47
200 °C	69.84	75.63	70.51	74.39	66.85	88.12	85.52	87.50	92.28
250 °C	71.36	75.53	73.29	68.85	68.10	86.00	86.03	80.14	84.08
300 °C	86.60	87.28	84.75	79.70	82.12	86.92	86.25	84.86	90.09
OA (%)	82.42	85.69	83.19	83.08	78.97	90.86	91.61	89.47	93.08
AA (%)	82.84	86.02	83.54	83.18	79.01	90.80	91.70	89.40	93.10
Kappa	0.7890	0.8283	0.7983	0.7970	0.7477	0.8903	0.8993	0.8737	0.9170

**Table 2:** The results after conducting ablation experiments on the bidirectional Mamba global spectral feature extraction module.

	Remove	Sequential	Reverse	Bidirectional
OA (%)	0.8892	92.44	92.83	93.08
AA (%)	0.8879	92.36	92.76	93.10
Kappa	0.8670	0.9033	0.9140	0.9170

**Table 3:** Processing time (in seconds) of each method.

	KNN	SVM	RF	GBM	LDA	MLP	1D CNN	Transformer	MBNet
Training	0.01	0.97	7.98	256.82	0.11	3.31	187.58	927.49	18.44
Prediction	0.21	1.30	0.03	0.03	0.01	0.01	0.06	0.46	0.02

## Data Availability

The data used in this study are available in the article.
